# Isolation, Genomic and Metabolomic Characterization of *Streptomyces tendae* VITAKN with Quorum Sensing Inhibitory Activity from Southern India

**DOI:** 10.3390/microorganisms8010121

**Published:** 2020-01-16

**Authors:** Nabila Mohammed Ishaque, Ilia Burgsdorf, Jessie James Limlingan Malit, Subhasish Saha, Roberta Teta, Daniela Ewe, Krishnan Kannabiran, Pavel Hrouzek, Laura Steindler, Valeria Costantino, Kumar Saurav

**Affiliations:** 1 Department of Biomedical Sciences, School of Biosciences and Technology, VIT University, Vellore 632014, India; nabilaimthiyaz@gmail.com (N.M.I.); kkb@vit.ac.in (K.K.); 2 Department of Marine Biology, Leon H. Charney School of Marine Sciences, University of Haifa, Mt. Carmel, Haifa 31905, Israel; burgsdorf84@gmail.com (I.B.); lsteindler@univ.haifa.ac.il (L.S.); 3 Department of Ocean Science, Division of Life Science and Hong Kong, Branch of the Southern Marine Science and Engineering Guangdong Laboratory, The Hong Kong University of Science and Technology, Hong Kong, China; jjmalit@connect.ust.hk; 4 Laboratory of Algal Biotechnology-Centre Algatech, Institute of Microbiology of the Czech Academy of Sciences, Opatovickýmlýn, Novohradská 237, 37981 Třeboň, Czech Republic; saha@alga.cz (S.S.); ewe@alga.cz (D.E.); hrouzek@alga.cz (P.H.); 5 The Blue Chemistry Lab, Dipartimento di Farmacia, Università degli Studi di Napoli Federico II, Via D. Montesano 49, 80131 Napoli, Italy; roberta.teta@unina.it

**Keywords:** natural product, actinobacteria, quorum sensing inhibition (QSI), biosynthetic gene clusters (BGCs), global natural product social networking (GNPS), cyclic dipeptides (2,5-diketopiperazines, DKPs), LC-HRMS

## Abstract

*Streptomyces* are among the most promising genera in terms of production ability to biosynthesize a variety of bioactive secondary metabolites with pharmaceutical interest. Coinciding with the increase in genomic sequencing of these bacteria, mining of their genomes for biosynthetic gene clusters (BGCs) has become a routine component of natural product discovery. Herein, we describe the isolation and characterization of a *Streptomyces tendae* VITAKN with quorum sensing inhibitory (QSI) activity that was isolated from southern coastal part of India. The nearly complete genome consists of 8,621,231bp with a GC content of 72.2%. Sequence similarity networks of the BGCs detected from this strain against the Minimum Information about a Biosynthetic Gene Cluster (MIBiG) database and 3365 BGCs predicted by antiSMASH analysis of publicly available complete *Streptomyces* genomes were generated through the BiG-SCAPE-CORASON platform to evaluate its biosynthetic novelty. Crude extract analysis using high-performance liquid chromatography connected to high resolution tandem mass spectrometry (HPLC-HRMS/MS) and dereplication through the Global Natural Product Social Molecular Networking (GNPS) online workflow resulted in the identification of cyclic dipeptides (2, 5-diketopiperazines, DKPs) in the extract, which are known to possess QSI activity. Our results highlight the potential of genome mining coupled with LC-HRMS/MS and in silico tools (GNPS) as a valid approach for the discovery of novel QSI lead compounds. This study also provides the biosynthetic diversity of BGCs and an assessment of the predicted chemical space yet to be discovered.

## 1. Introduction

Natural products of microbial origin have gained considerable attention because of their diverse chemical structures and bioactivity representing almost 35% of all present drugs [1,2]. Our current use of antibiotics poses great challenges among medical practitioners and researchers due to the development and spread of antibiotic–resistant pathogens. Moreover, the low success rate of antibiotic drug discovery programs worldwide has resulted in today’s eminent lack of new antibiotic drug leads [3]. An increased understanding on the mechanism of bacterial pathogenesis and intercellular microbial communication has revealed potential alternative strategies to treat bacteria-mediated diseases. A crucial aspect for the establishment and maintenance of a microbial population is based on cell–cell communication, which triggers genetic regulation based on cell density, thus coordinating the physiologies of the different cell types through a mechanism known as quorum sensing (QS) [4]. Autoinducers (AIs) are diverse small secreted signaling molecules whose elicited responses can contribute directly to pathogenesis through the synchronized production of various virulence determinants [5]. Additionally, it is theorized that blocking the bacterial signal communication would disable its ability to mount an organized assault on the host and thus will prevent the development of resistance [6,7].

Initial screening and discovery for novel lead molecules is usually performed at the level of crude extract screening followed by bioactivity-guided fractionation and purification [8]. However, this approach suffers drawback due to large sample complexity resulting in additive or multiplicative effects of the compounds present in the extract together with potential rediscovery of already known compounds or its analogs. This can be partially overcome by pre-fractionation of the crude extracts, coupled with an advanced dereplication approach through the use of fast and extremely accurate methods such as high-performance liquid chromatography connected to high resolution tandem mass spectrometry (HPLC-HRMS/MS) in the field of natural products to develop high-throughput discovery pipelines [9,10]. Another drawback is the presence of cryptic genes disabling the production/detection of desired bioactive lead molecules using the conventional way. The presence of these cryptic biosynthetic gene clusters (BGCs) could be viewed by genome sequencing. Based on antiSMASH analysis, it is now apparent that many bacteria with large genomes, particularly actinomycetes, have the coding capacity to produce many secondary metabolites such as antibiotic, antitumor, and immunomodulating drugs [11,12]. In our continuing effort to isolate new bioactive molecules [2,13], it is crucial that new groups of actinomycetes from unexplored places or under exploited conditions be pursued as sources of novel bioactive secondary metabolites. Herein, we report the isolation of *Streptomyces tendae* VITAKN with quorum sensing inhibitory (QSI) activity from a southern coastal area of India, together with its almost complete (18 scaffolds, 100% completeness, and 0% heterogeneity) draft genome sequence.

## 2. Materials and Methods

### 2.1. Isolation of Actinomycetes

The marine soil sample was aseptically collected from the Rameswaram coast (9.2876° N, 79.3129° E), Tamil Nadu, India at a depth of 10–15 cm in sterile polythene bags and transported to the laboratory. The pre-treatment of the soil sample was done in a hot air oven at 70 °C for 30 min following the previously described protocol with slight modification [14,15] and the sample was serially diluted up to 10^−6^ dilution before plated on Actinomycetes Isolation Agar (AIA) (sodium caseinate, 2.0 g/L; l-Asparagine, 0.1 g/L; sodium propionate,4.0 g/L; dipotassium phosphate, 0.5 g/L; magnesium sulfate, 0.1 g/L; ferrous sulfate, 0.001 g/L; and agar, 15 g/L, adjusted to a final pH of 8.1 ± 0.2) and Starch Casein Agar (SCA) (starch, 10 g/L; casein powder, 1 g/L; sea water, 37 g/L; and agar, 15 g/L, adjusted to a final pH of 7.2 ± 0.2) (HiMedia Laboratories, Mumbai, India). The plates were incubated at room temperature for 7–14 days. Isolated actinomycetes were sequentially sub-cultured on AIA plates until pure culture was obtained. Pure culture was maintained at 28 °C until future use.

### 2.2. Morphological and Cultural Characteristics of the Strain

The isolate, VITAKN was further identified using various cultural characteristics including the growth optimization parameters on different media with (3% *w/v*) sea salt; The International *Streptomyces* Project (ISP) medium 1 (Tryptophan Yeast Extract Broth), ISP2 (Yeast Malt Agar), ISP3 (Oat Meal Agar), ISP4 (Inorganic Salt Starch Agar), ISP5 (Glycerol Asparagine Agar Base), ISP6 (Peptone Yeast Extract Iron HiVeg Agar), ISP7 (Tyrosine Agar Base), ISP9 (Carbon Utilization Agar), AIA, SCA, and NA (Nutrient Agar) (HiMedia Laboratories, Mumbai, India); temperature (30, 40, and 50 °C); and pH (4, 6, 7, 9, and 12). Genus level identification of the isolate was carried out based on aerial and substrate mycelium, reverse side pigmentation, and spore chain morphology following the Bergey’s Manual of Determinative Bacteriology. The arrangement of the spores in the mycelium was observed by cover slip method under light microscope and by scanning electron microscope. Growth pattern was also evaluated on different carbon supplements (1% glucose, fructose, maltose, mannitol, and starch) using ISP9 medium [16].

### 2.3. Crude Extracts Preparation and Quorum Sensing Inhibitory (QSI) Activity

Pure culture of the strain was further inoculated in small scale fermentation AM3 medium (soluble starch, 15 g/L; soybean powder, 5 g/L; peptone bacteriological, 15 g/L; glycerol, 15 g/L; CaCO_3_, 2 g/L; 3% sea salt; and pH 7.4). After one week of incubation, the crude extract was prepared by thrice extracting the cell free supernatant with ethyl acetate and drying in vacuo to obtain 12.5–0.0487 mg/mL in 20% DMSO in water. QSI activity was tested using luminescence-based reporters, *E. coli* pSB401 and *E. coli* pSB1075, and was quantified on a TriStar Multimode Microplate reader (Berthold Technologies GmbH & Co. KG, Bad Wildbad, Germany) following the authors of [17,18]. The plasmids pSB401 and pSB1075 contain the *luxI/R* and *lasI/R* gene promoters, respectively, regulate *luxCDABE* expression, and respond well to exogenously provided Acyl Homoserine Lactones (AHLs) [17,19]. Overnight cultures of the reporter strains were diluted to OD_600_ of 0.01 and 50 µL was added to each well of 96-well plates. Addition of *N*-(3-oxo-hexanoyl)-l-homoserine lactone (3-oxo-C6-HSL, 1 μm final concentration) and *N*-(3-oxo-dodecanoyl)-l-homoserine lactone (3-oxo-C12-HSL, 2 μm final concentration) stimulated the QS activity of pSB401 and pSB1075 biosensors, respectively. Bioluminescence was measured after 4 h. Non-inhibitory concentration for the crude extract dilutions against both the biosensor without the exogenously addition of its cognate HSL were also determined.

### 2.4. Genomic Characterization

Strain VITAKN, was grown on ISP1 medium for four days (HiMedia, Mumbai, India) to obtain the mycelium for DNA isolation. Genomic DNA was isolated using the High pure PCR template Preparation Kit, Roche following manufacturer’s protocol. DNA libraries were prepared using the TruSeq DNA PCR free kit. Metagenomic shotgun libraries were sequenced on an Illumina platform (100-bp paired-end reads) in Macrogen, Korea, yielding a total of 6.6G bp of sequence. Sequences were trimmed using Trimmomatic version 0.36 (minlen 100, sliding window 4:20) [20], and read quality was assessed using FastQC version 0.11.5 [21]. Prior to de novo assembly using SPADES version 3.13.0 (parameters: −*k* 21,33,55,77,91-careful-only-assembler-cov-cutoff auto) [22]. Scaffolds ≥2 kb were used for further analysis. Completeness and contamination were estimated with checkM version 1.0.7 [23] based on 460 markers and using taxonomy workflow for *Streptomycetaceae*. The near-full-length 16S rRNA gene sequence was obtained as follows: First, rRNA sequences were sorted from the raw shotgun readsusingSortMeRNA (v. 2.1b) [24] and SILVA 132 as reference database. Second, the obtained reads were assembled with rnaSPAdes [25] using default parameters. SILVA 132 non-redundant databases were downloaded from the SILVA official website (http://www.arb-silva.de). For the phylogenetic analysis, sequences of the closest described type strains were obtained from EzTaxon [26]. Open reading frames (ORFs) for AKN genome were identified and annotated with the RASTtk algorithm [27,28]. The sequences were compared with those available in Genbank using BLASTn [29]. Putative cyclodipeptide synthases (CDPS) were predicted using HMMER version 3.2.1 [30]. Model (HMM) profile of CDPS (PF16715) was downloaded from the PFAM website (https://pfam.xfam.org/).

To further assess the biosynthetic capacity of this strain, antiSMASH v4.1 [31] was used to identify the BGCs encoded in its genome, along with 113 other publicly available complete *Streptomyces* RefSeq genomes obtained from the National Center for Biotechnology Information. BGCs were then clustered into groups based on sequence similarity using BiG-SCAPE [32] using default parameters, including singletons. A distance cut-off score of 0.3. MIBiG database v1.4 [33] was also applied to the networks to assign BGCs that putatively produce known compounds. Generated networks were visualized through an open source software platform, Cytoscape (version 3.7.2) [34].

### 2.5. HPLC-HRMS/MS Analysis and Metabolites Identification Using GNPS Dereplication

Crude extract was analyzed by LC-HRMS and LC-HRMS/MS using a Thermo LTQ Orbitrap XL mass spectrometer (Thermo Fisher Scientific Spa, Rodano, Italy) coupled to an Agilent model 1100 LC system (Agilent Technologies, CernuscosulNaviglio, Italy) equipped with a solvent reservoir, an in-line degasser, a binary pump, and a refrigerated autosampler [13]. The spectrum was recorded by infusion into the ESI source using MeOH as the solvent. A 5 μm Kinetex C18 column (50 mm × 2.1 mm), maintained at 25 °C, was operated using a gradient elution of H_2_O and MeOH both with 0.1% formic acid, running at 200 μL/min. The gradient program was as follows: 10% MeOH for 3 min, 10–90% MeOH over 30 min, and 90% MeOH for 3 min. Data dependent acquisition mode MS spectra was recorded in positive ion mode with a spray voltage of 5 kV, a capillary temperature of 230 °C, a sheath gas rate of 12 units N_2_ (ca. 120 mL/min), and an auxiliary gas rate of 5 units N_2_ (ca. 50 mL/min). HRMS/MS scans were obtained for selected ions with collision-induced dissociation (CID) fragmentation, isolation width of 2.0, normalized collision energy of 35, activation Q of 0.250, and activation time of 30 ms. A molecular network was created with the Feature-Based Molecular Networking (FBMN) workflow [35] on Global Natural Product Social networking (GNPS) (https://gnps.ucsd.edu) [36,37]. The mass spectrometry data were first processed with MZMINE v2.52 [38] and the results were exported to GNPS for FBMN analysis. The data were filtered by removing all MS/MS fragment ions within ±17 Da of the precursor *m*/*z*. MS/MS spectra were window filtered by choosing only the top 6 fragment ions in the ±50 Da window throughout the spectrum. A molecular network was then created and the spectra in the network were searched against GNPS spectral libraries [36,39]. The DEREPLICATOR was used to annotate and identify metabolites through the database search of MS/MS spectra [10]. The molecular networks were visualized using Cytoscape software v3.7.2 [34].

### 2.6. Data Deposition and Job Accessibility 

The mass spectrometry data were deposited on MassIVE public repository (MSV000084793). The molecular networking job can be publicly accessed at: https://gnps.ucsd.edu/ProteoSAFe/status.jsp?task=2f692735adc7493c84080eb3042fadb5. The sequence of the putative CDPS gene was deposited in the NCBI, GenBank database under accession No. MN927583. The draft genome of the VITAKN has been deposited under BiosampleNo. SUB6814882 (Bioproject No. PRJNA600621).

## 3. Results and Discussion

**Figure 1:**
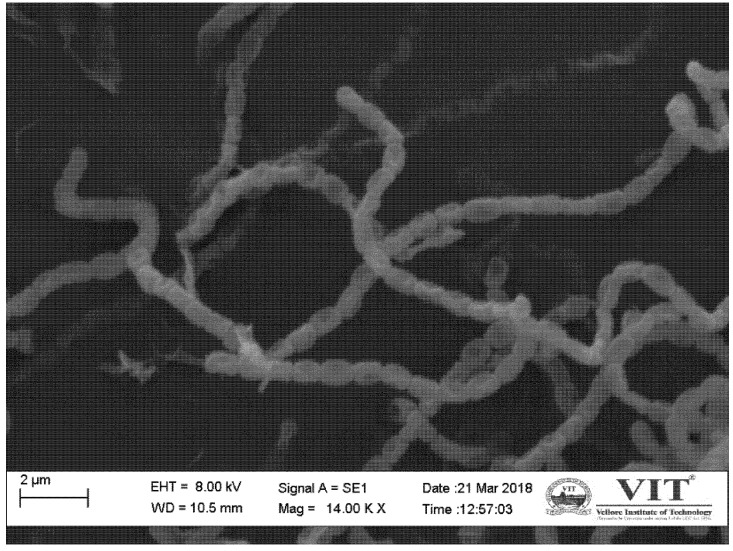
Image of strain VITAKN on scanning electron microscopy.

The isolate VITAKN produced white powdery, raised small to medium sized colonies, while the substrate mycelium was pale white in color when cultured on AIA plates. The spores had a smooth surface, oblong in shape and were arranged in chains when examined using a scanning electron microscope (1000× magnification) (Figure 1), suggesting that the isolate might belong to *Streptomyces*. The genus, *Streptomyces*, can be easily distinguished from other bacterial groups based on its distinctive morphological, physiological, and biochemical features [40]. Hence, the isolate VITAKN was further characterized by the methods recommended by the ISP. The growth of the isolate on different media is given in Table S1. The isolate VITAKN grew abundantly on ISP1, ISP2, ISP4, and ISP7, whereas good to moderate growth was observed once grown on ISP3, ISP5, ISP6, NA, and SCA. The isolate VITAKN showed positive for Gram’s staining test, methyl red test, and citrate utilization test and showed negative for mannitol test, indole, and Voges Proskauer’s test. It showed an alkaline butt with alkaline slant in the triple-sugar iron test. The isolate was non-motile and found to be positive for lipase, oxidase, catalase, and urease and negative for amylase (Table S2). The cultural condition for the growth of the isolate was optimized. The isolate grew very well in up to 4% NaCl concentration, moderate growth in 6% NaCl, and no growth was observed in 10% NaCl. The isolate growth was excellent at pH 7 and moderate growth was observed at pH 6 or 9. Based on the temperature, the isolate had very good growth between 28 and 30 °C. It grew moderately at 40 °C with no growth at 50 °C. Combining all the obtained morphological, microscopical, physiological, and biochemical properties, they clearly confirmed that VITAKN strain belongs to genus *Streptomyces*.

Non-inhibitory concentration (NIC) was determined against pSB401 and pSB1075 perquisite to QSI activity to rule out artifacts caused by growth inhibition. No inhibitory activities were observed at concentrations of 0.0487–12.5 mg/mL. Crude extracts inhibited luminescence by 20% at 0.78125 mg/mL (for pSB401and pSB1075). These data suggest the presence of some QSI active molecule in the extract and hence required further investigation (Figure S1).

**Figure 2:**
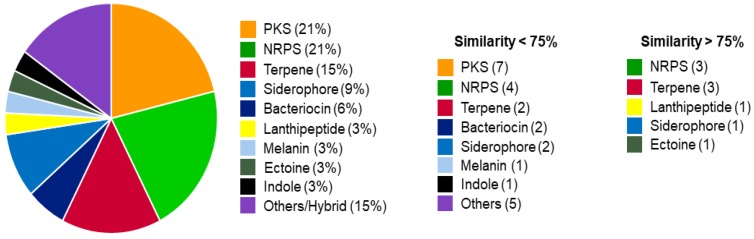
Biosynthetic gene clusters predicted from this strain using antiSMASH. Out of the 33 clusters identified, 24 clusters possess genes with less than 75% similarity to that of known compounds.

**Figure 3:**
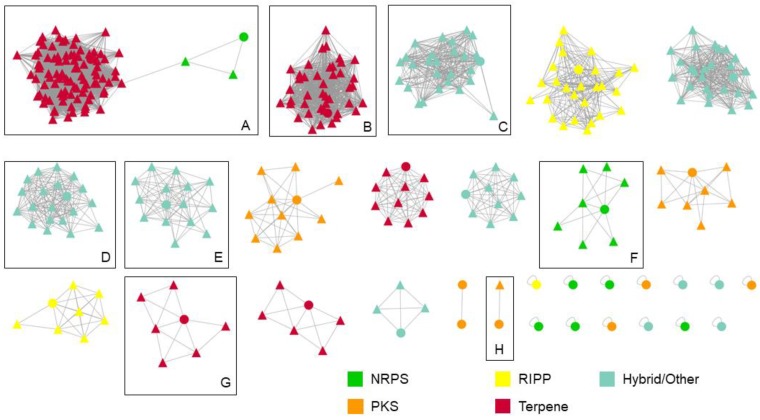
Sequence similarity network of the 33 biosynthetic gene clusters detected in *S. tendae* VITAKNcompared against BGCs in the MIBiG database and other *Streptomyces* strains. Circular nodes represent BGCs from this strain, and triangular nodes represent MIBiG and other *Streptomyces* BGCs. Boxed clusters represent clusters containing nodes associated with a MIBiG BGC: (**A**) coelibactin and hopene; (**B**) albaflavenone; (**C**) ectoine; (**D**) desferrioxamin; (**E**) coelibactin; (**F**) coelichelin; (**G**) isorenieratene; and (**H**) griseusin. Colors were schemed according to different BGC family annotation.

VITAKN genome assembly yielded 20 scaffolds with an *N*_50_ value of 788,548 bp, the largest contig having 1,671,513 bp. The genome consists of 8,621,231 bp with a GC content of 72.2%with 100% estimated completeness, 0.06% contamination and 0% heterogeneity. The AKN genome contains 7898 predicted and 69 RNA genes (67 tRNA genes). The 16S rRNA gene was assembled separately (see Section 2.4). Based on EzTaxon analyses of the extracted 16S rRNA gene (1564bp), the strain showed 99.8% similarity to *Streptomyces tendae* ATCC 19,812 and hence strain AKN was designated as *Streptomycestendae* VITAKN. antiSMASH analysis identified 33 clusters, mostly encoding for polyketide synthases (PKS) and hybrid-PKS (12) and non-ribosomal peptide synthases (NRPS) (7) BGCs, both of which are well known for producing structurally diverse compounds displaying various bioactivity. Detected BGCs belonging to other classes include five terpenes, two siderophores, two bacteriocins, and one of each of the following: melanin, ectoine, lanthipeptide, indole, and an unassigned cluster (Figure 2). In addition, nine clusters possess sequence similarity with more than 75% to known BGCs. Interestingly, two BGCs showed 100% similarity with SapB (a lantibiotic-like peptide) and coelichelin (siderophore), both known to be produced by *Streptomyces coelicolor*. SapB is a morphogenetic peptide that is important for aerial mycelium formation by the filamentous bacterium *Streptomyces coelicolor* [41]. Another BGC encoding for coelibactin, a NRPS synthesized peptide with predicted zincophore activity, has been implicated in antibiotic regulation in *Streptomyces coelicolor* A3 (2) [42]. Manual curation of antiSMASH data inspired us to mine the genome to explore and exploit BGCs diversity to larger extent. These BGCs can be readily identified in a genome sequence as they are usually clustered together on the chromosome. The novelty and uniqueness of the BGCs predicted from this strain was evaluated through sequence similarity analysis with the MIBiG database and 3365 existing BGCs predicted by antiSMASH analysis of publicly available complete *Streptomyces* genomes. The genus *Streptomyces* is one of the most chemically exploited genera for discovering novel drugs since the discovery of penicillin and streptomycin. Further, the establishment of full genome sequences of two well studied natural product producing strains, *Streptomyces coelicolor* [43] and *Streptomyces avermitilis* [44], led scientists to notice the unexplored potential hidden in bacterial genomes [45]. A *Streptomyces* genome contains on average about 30 secondary metabolite gene clusters, but the limitation of analytic detection inspired us to explore the strain in depth and suggested that even well studied strains contain the genetic potential to synthesize more compounds than detected analytically. One of the largest manually annotated BGC collections, MIBiG, provided a highly curated reference dataset (1920 BGCs). Out of all the BGCs curated, 636 BGCs have been entered from genus *Streptomyces* alone followed by *Aspergillus* (88 BGCs) and *Pseudomonas* (68 BGCs) [46]. This survey allowed us to assess the potential of discovering novel metabolites from the isolated *S.* *tendae* VITAKN and to depict unexplored biosynthetic space. Out of 33 BGCs observed from the strain, only nine BGCs displayed similarity with known BGCs, and only 17 BGCs associated with other BGCs extracted from existing *Streptomyces* genomes (Figure 3), warranting further exploration of the biosynthetic potential of this strain for producing novel bioactive secondary metabolites.

The chemical structures of their products can be predicted to a certain extent, based on the analysis and biosynthetic logic of the enzymes encoded in a BGC and their similarity to known counterparts [47]. However, it is rare to detect these compounds putatively encoded by these BGCs analytically. However, we analyzed the crude extract of *S. tendae* VITAKN for metabolites by LC-HRMS and LC-HRMS/MS. This was done to determine whether the extract containsunique chemical diversity and to test if these can be matched with the genetically encoded BGCs. To create a molecular network, the LC-MS/MS data were analyzed and dereplicated against publicly available datasets accessed via the GNPS-MassIVE database. To date, >93 million MS/MS spectra from various instruments (e.g., Orbitrap and qTOF) have been searched at GNPS, yielding putative dereplication matches of 7.7 million spectra to 15,477 compounds [36,48]. The usage of this platform yielded the formation of the molecular network containing 327 parent ions (nodes) (Figure S2).

**Figure 4:**
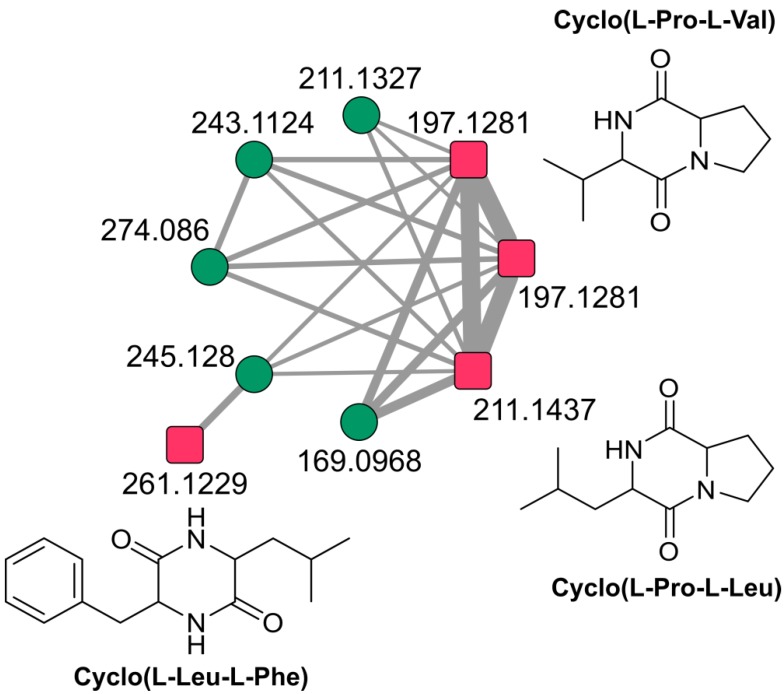
2, 5-diketopiperazines cluster in *S. tendae* VITAKN molecular network. Each node represents *m*/*z* value of the parent ion and edge thickness signifies cosine score similarity.

These metabolites formed 24 clusters and 186 self-loop nodes. High throughput molecular networking in our study showed putative presence of cyclic dipeptides (2,5-diketopiperazines, DKPs) in Cluster 4. The four cyclic peptides identified using the database in our present study are cyclo-(Leu-Phe), cyclo-(l-Leu-l-Pro), cyclo-(l-Pro-l-Val), and cyclo-(L-Trp-L-Pro) (Figure 4). These DKPs have been previously reported to activate or inhibit LuxR-type proteins in AHL biosensor strains, albeit at significantly higher concentrations than native lactones [49]. DKPs are a class of compounds, which have gained considerable interest in recent years and have been investigated as potential anticancer, antibiotics, and anti-inflammatory agents [50]. The biosynthesis of 2,5-diketopiperazines are mediated by a family of enzymes known as CDPS [51]. CDPS catalyzes the formation of cyclodipeptides using aninoacylated-tRNA as substrate. Further, we looked for the putative CDPS in our genome and the putative CDPS protein VITAKN_3870 was predicted with HMM search (*E* value = 0.026). These finding further emphasize to continue our effort on isolating and purifying these molecules and identifying their exact activity, as well as identifying other molecules present in the clusters that can possibly be new analogs of these DKPs. High throughput FBMN in our study did not match with the large datasets of the GNPS repository, indicating the putative presence of a new class of compounds or the absence of produced compounds from repository.

This strain may produce many yet unknown compounds, one of which may be the relevant for the observed QSI activity apart from detected DKPs in the extract. Furthermore, the use of DEREPLICATOR as part of the library search for in silico identification of both peptidic and non-peptidic natural productsis an advanced tool to identify the known among unknown [9,10].

## 4. Conclusions

The continued rise in antibiotic resistance needs to be addressed by discovering an alternative way for tackling infections. QSI is one of many strategies being investigated. Moreover, exploration of novel isolates continues to be important for the exploitation of new metabolic potential. The crude extract from the strain *S. tendae* VITAKN isolated from southern Indian soil showed QSI activity, whereas genome sequencing and subsequent BGCs analysis showed the presence of diverse BGCs after comparison with other BGCs from other *Streptomyces* species. The present study also used chemical fingerprinting and molecular networking to identify the presence of metabolites in the crude extract. These results warrant further exploration of this strain for the production of novel bioactive secondary metabolites.

## Supplementary Materials

Supplementary materials can be found at https://www.mdpi.com/2076-2607/8/1/121/s1.

## References

[1] Benndorf R., Guo H., Sommerwerk E., Weigel C., Garcia-Altares M., Martin K., Hu H., Kufner M., de Beer Z.W., Poulsen M. (2018). Natural Products from Actinobacteria Associated with Fungus-Growing Termites. Antibiotics.

[2] Teta R., Marteinsson V.T., Longeon A., Klonowski A.M., Groben R., Bourguet-Kondracki M.L., Costantino V., Mangoni A. (2017). Thermoactinoamide A, an Antibiotic Lipophilic Cyclopeptide from the Icelandic Thermophilic Bacterium Thermoactinomyces vulgaris. J. Nat. Prod..

[3] Chokshi A., Sifri Z., Cennimo D., Horng H. (2019). Global Contributors to Antibiotic Resistance. J. Glob. Infect. Dis..

[4] Waters C.M., Bassler B.L. (2005). Quorum sensing: Cell-to-cell communication in bacteria. Annu. Rev. Cell Dev. Biol..

[5] LaSarre B., Federle M.J. (2013). Exploiting quorum sensing to confuse bacterial pathogens. Microbiol. Mol. Biol. Rev..

[6] Saurav K., Bar-Shalom R., Haber M., Burgsdorf I., Oliviero G., Costantino V., Morgenstern D., Steindler L. (2016). In Search of Alternative Antibiotic Drugs: Quorum-Quenching Activity in Sponges and their Bacterial Isolates. Front. Microbiol..

[7] Della Sala G., Teta R., Esposito G., Costantino V., Tommonaro G. (2019). Chapter 1—The Chemical Language of Gram-Negative Bacteria. Quorum Sensing.

[8] Strömstedt A.A., Felth J., Bohlin L. (2014). Bioassays in Natural Product Research—Strategies and Methods in the Search for Anti-inflammatory and Antimicrobial Activity. Phytochem. Anal..

[9] Mohimani H., Gurevich A., Mikheenko A., Garg N., Nothias L.F., Ninomiya A., Takada K., Dorrestein P.C., Pevzner P.A. (2017). Dereplication of peptidic natural products through database search of mass spectra. Nat. Chem. Biol..

[10] Mohimani H., Gurevich A., Shlemov A., Mikheenko A., Korobeynikov A., Cao L., Shcherbin E., Nothias L.F., Dorrestein P.C., Pevzner P.A. (2018). Dereplication of microbial metabolites through database search of mass spectra. Nat. Commun..

[11] Schorn M.A., Alanjary M.M., Aguinaldo K., Korobeynikov A., Podell S., Patin N., Lincecum T., Jensen P.R., Ziemert N., Moore B.S. (2016). Sequencing rare marine actinomycete genomes reveals high density of unique natural product biosynthetic gene clusters. Microbiology.

[12] Carroll A.R., Copp B.R., Davis R.A., Keyzers R.A., Prinsep M.R. (2019). Marine natural products. Nat. Prod. Rep..

[13] Esposito G., Bourguet-Kondracki M.L., Mai L.H., Longeon A., Teta R., Meijer L., Van Soest R., Mangoni A., Costantino V. (2016). Chloromethylhalicyclamine B, a Marine-Derived Protein Kinase CK1delta/epsilon Inhibitor. J. Nat. Prod..

[14] Gebreyohannes G., Moges F., Sahile S., Raja N. (2013). Isolation and characterization of potential antibiotic producing actinomycetes from water and sediments of Lake Tana, Ethiopia. Asian Pac. J. Trop. Biomed..

[15] Hong K., Gao A.H., Xie Q.Y., Gao H., Zhuang L., Lin H.P., Yu H.P., Li J., Yao X.S., Goodfellow M. (2009). Actinomycetes for marine drug discovery isolated from mangrove soils and plants in China. Mar. Drugs.

[16] Kumar S., Kannabiran K. (2010). Antifungal activity of Streptomyces VITSVK5 spp. against drug resistant Aspergillus clinical isolates from pulmonary tuberculosis patients. J. Mycol. Med..

[17] Winson M.K., Swift S., Fish L., Throup J.P., Jorgensen F., Chhabra S.R., Bycroft B.W., Williams P., Stewart G.S. (1998). Construction and analysis of luxCDABE-based plasmid sensors for investigating *N*-acyl homoserine lactone-mediated quorum sensing. FEMS Microbiol. Lett..

[18] Saurav K., Costantino V., Venturi V., Steindler L. (2017). Quorum Sensing Inhibitors from the Sea Discovered Using Bacterial N-acyl-homoserine Lactone-Based Biosensors. Mar. Drugs.

[19] Costantino V., Della Sala G., Saurav K., Teta R., Bar-Shalom R., Mangoni A., Steindler L. (2017). Plakofuranolactone as a Quorum Quenching Agent from the Indonesian Sponge Plakortis cf. lita. Mar. Drugs.

[20] Bolger A.M., Lohse M., Usadel B. (2014). Trimmomatic: A flexible trimmer for Illumina sequence data. Bioinformatics.

[21] Wingett S.W., Andrews S. (2018). FastQ Screen: A tool for multi-genome mapping and quality control. F1000Res..

[22] Bankevich A., Nurk S., Antipov D., Gurevich A.A., Dvorkin M., Kulikov A.S., Lesin V.M., Nikolenko S.I., Pham S., Prjibelski A.D. (2012). SPAdes: A new genome assembly algorithm and its applications to single-cell sequencing. J. Comput. Biol..

[23] Parks D.H., Imelfort M., Skennerton C.T., Hugenholtz P., Tyson G.W. (2015). CheckM: Assessing the quality of microbial genomes recovered from isolates, single cells, and metagenomes. Genome Res..

[24] Kopylova E., Noe L., Touzet H. (2012). SortMeRNA: Fast and accurate filtering of ribosomal RNAs in metatranscriptomic data. Bioinformatics.

[25] Bushmanova E., Antipov D., Lapidus A., Prjibelski A.D. (2019). rnaSPAdes: A de novo transcriptome assembler and its application to RNA-Seq data. Gigascience.

[26] Kim O.S., Cho Y.J., Lee K., Yoon S.H., Kim M., Na H., Park S.C., Jeon Y.S., Lee J.H., Yi H. (2012). Introducing EzTaxon-e: A prokaryotic 16S rRNA gene sequence database with phylotypes that represent uncultured species. Int. J. Syst. Evol. Microbiol..

[27] Brettin T., Davis J.J., Disz T., Edwards R.A., Gerdes S., Olsen G.J., Olson R., Overbeek R., Parrello B., Pusch G.D. (2015). RASTtk: A modular and extensible implementation of the RAST algorithm for building custom annotation pipelines and annotating batches of genomes. Sci. Rep..

[28] Overbeek R., Olson R., Pusch G.D., Olsen G.J., Davis J.J., Disz T., Edwards R.A., Gerdes S., Parrello B., Shukla M. (2014). The SEED and the Rapid Annotation of microbial genomes using Subsystems Technology (RAST). Nucleic Acids Res..

[29] Altschul S.F., Madden T.L., Schaffer A.A., Zhang J., Zhang Z., Miller W., Lipman D.J. (1997). Gapped BLAST and PSI-BLAST: A new generation of protein database search programs. Nucleic Acids Res..

[30] Eddy S.R. (2011). Accelerated Profile HMM Searches. PLoSComput. Biol..

[31] Blin K., Wolf T., Chevrette M.G., Lu X., Schwalen C.J., Kautsar S.A., Suarez Duran H.G., de Los Santos E.L.C., Kim H.U., Nave M. (2017). antiSMASH 4.0-improvements in chemistry prediction and gene cluster boundary identification. Nucleic Acids Res..

[32] Navarro-Muñoz J.C., Selem-Mojica N., Mullowney M.W., Kautsar S.A., Tryon J.H., Parkinson E.I., De Los Santos E.L.C., Yeong M., Cruz-Morales P., Abubucker S. (2020). A computational framework to explore large-scale biosynthetic diversity. Nat. Chem. Biol..

[33] Medema M.H., Kottmann R., Yilmaz P., Cummings M., Biggins J.B., Blin K., de Bruijn I., Chooi Y.H., Claesen J., Coates R.C. (2015). Minimum Information about a Biosynthetic Gene cluster. Nat. Chem. Biol..

[34] Shannon P., Markiel A., Ozier O., Baliga N.S., Wang J.T., Ramage D., Amin N., Schwikowski B., Ideker T. (2003). Cytoscape: A software environment for integrated models of biomolecular interaction networks. Genome Res..

[35] Nothias L.F., Petras D., Schmid R., Dührkop K., Rainer J., Sarvepalli A., Protsyuk I., Ernst M., Tsugawa H., Fleischauer M. (2019). Feature-based Molecular Networking in the GNPS Analysis Environment. bioRxiv.

[36] Wang M., Carver J.J., Phelan V.V., Sanchez L.M., Garg N., Peng Y., Nguyen D.D., Watrous J., Kapono C.A., Luzzatto-Knaan T. (2016). Sharing and community curation of mass spectrometry data with Global Natural Products Social Molecular Networking. Nat. Biotechnol..

[37] Caso A., Esposito G., Della Sala G., Pawlik J.R., Teta R., Mangoni A., Costantino V. (2019). Fast Detection of Two Smenamide Family Members Using Molecular Networking. Mar. Drugs.

[38] Pluskal T., Castillo S., Villar-Briones A., Oresic M. (2010). MZmine 2: Modular framework for processing, visualizing, and analyzing mass spectrometry-based molecular profile data. BMC Bioinform..

[39] Horai H., Arita M., Kanaya S., Nihei Y., Ikeda T., Suwa K., Ojima Y., Tanaka K., Tanaka S., Aoshima K. (2010). MassBank: A public repository for sharing mass spectral data for life sciences. J. Mass Spectrom..

[40] Shirling E.B., Gottlieb D. (1966). Methods for characterization of Streptomyces species1. Int. J. Syst. Evol. Microbiol..

[41] Kodani S., Hudson M.E., Durrant M.C., Buttner M.J., Nodwell J.R., Willey J.M. (2004). The SapB morphogen is a lantibiotic-like peptide derived from the product of the developmental gene ramS in Streptomyces coelicolor. Proc. Natl. Acad. Sci. USA.

[42] Zhao B., Moody S.C., Hider R.C., Lei L., Kelly S.L., Waterman M.R., Lamb D.C. (2012). Structural analysis of cytochrome P450 105N1 involved in the biosynthesis of the zincophore, coelibactin. Int. J. Mol. Sci..

[43] Bentley S.D., Chater K.F., Cerdeño-Tárraga A.M., Challis G.L., Thomson N.R., James K.D., Harris D.E., Quail M.A., Kieser H., Harper D. (2002). Complete genome sequence of the model actinomycete Streptomyces coelicolor A3(2). Nature.

[44] Ikeda H., Ishikawa J., Hanamoto A., Shinose M., Kikuchi H., Shiba T., Sakaki Y., Hattori M., Omura S. (2003). Complete genome sequence and comparative analysis of the industrial microorganism Streptomyces avermitilis. Nat. Biotechnol..

[45] Ziemert N., Alanjary M., Weber T. (2016). The evolution of genome mining in microbes—A review. Nat. Prod. Rep..

[46] Kautsar S.A., Blin K., Shaw S., Navarro-Muñoz J.C., Terlouw B.R., van der Hooft J.J.J., van Santen J.A., Tracanna V., Suarez Duran H.G., Pascal Andreu V. (2019). MIBiG 2.0: A repository for biosynthetic gene clusters of known function. Nucleic Acids Res..

[47] Medema M.H., Fischbach M.A. (2015). Computational approaches to natural product discovery. Nat. Chem. Biol..

[48] Frank A.M., Bandeira N., Shen Z., Tanner S., Briggs S.P., Smith R.D., Pevzner P.A. (2008). Clustering millions of tandem mass spectra. J. Proteome Res..

[49] Campbell J., Lin Q., Geske G.D., Blackwell H.E. (2009). New and unexpected insights into the modulation of LuxR-type quorum sensing by cyclic dipeptides. ACS Chem. Biol..

[50] Yao T., Liu J., Liu Z., Li T., Li H., Che Q., Zhu T., Li D., Gu Q., Li W. (2018). Genome mining of cyclodipeptide synthases unravels unusual tRNA-dependent diketopiperazine-terpene biosynthetic machinery. Nat. Commun..

[51] Canu N., Moutiez M., Belin P., Gondry M. (2019). Cyclodipeptide synthases: A promising biotechnological tool for the synthesis of diverse 2,5-diketopiperazines. Nat. Prod. Rep..

