# Supplementary material for: Isolation, Genomic and Metabolomic Characterization of Streptomyces tendae VITAKN with Quorum Sensing Inhibitory Activity from Southern India
Source: Microorganisms. 2020 Jan 16;8(1):121. doi: 10.3390/microorganisms8010121 (PMC7023471; doi:10.3390/microorganisms8010121)
Supplement: Supplementary file 1 [file microorganisms-08-00121-s001.zip › microorganisms-695040-supplementary 3/Supplementary_R1.docx]

**Isolation, genomic and metabolomic characterization of *Streptomyces tendae* VITAKN with quorum sensing inhibitory activity from southern India**

Nabila Mohammed Ishaque^1^, Ilia Burgsdorf^2^, Jessie James LimlinganMalit^3^, SubhasishSaha^4^, Roberta Teta^5^, Daniela Ewe^4^, Krishnan Kannabiran^1^, Pavel Hrouzek^4^, Laura Steindler^2^,
Valeria Costantino^5,^* and Kumar Saurav^1,2,4,5,^*

^1^ Department of Biomedical Sciences, School of Biosciences and Technology, VIT University, Vellore 632014, India; nabilaimthiyaz@gmail.com (N.M.I.); kkb@vit.ac.in (K.K.)

^2^ Department of Marine Biology, Leon H. Charney School of Marine Sciences, University of Haifa, Mt. Carmel, Haifa 31905, Israel; burgsdorf84@gmail.com (I.B.); lsteindler@univ.haifa.ac.il (L.S.)

^3^ Department of Ocean Science, Division of Life Science and Hong Kong, Branch of the Southern Marine Science and Engineering Guangdong Laboratory, The Hong Kong University of Science and Technology, Hong Kong, China; jjmalit@connect.ust.hk (J.M.)

^4^ Laboratory of Algal Biotechnology-Centre Algatech, Institute of Microbiology of the Czech Academy of Sciences, Opatovickýmlýn, Novohradská 237, 37981Třeboň, Czech Republic; saha@alga.cz (S.S.); ewe@alga.cz (D.E.); hrouzek@alga.cz (P.H.)

^5^ The Blue Chemistry Lab, Dipartimento di Farmacia, UniversitàdegliStudi di Napoli Federico II, Via D. Montesano 49, 80131 Napoli, Italy; roberta.teta@unina.it (R.T.)

***** Correspondence: valeria.costantino@unina.it (V.C.); sauravverma17@gmail.com (K.S.); Tel.: +39-0-8167-8504 (V.C.); +420-3-8434-0469 (K.S.)

Received: 30 December 2019; Accepted: 13 January 2020; Published: date

**
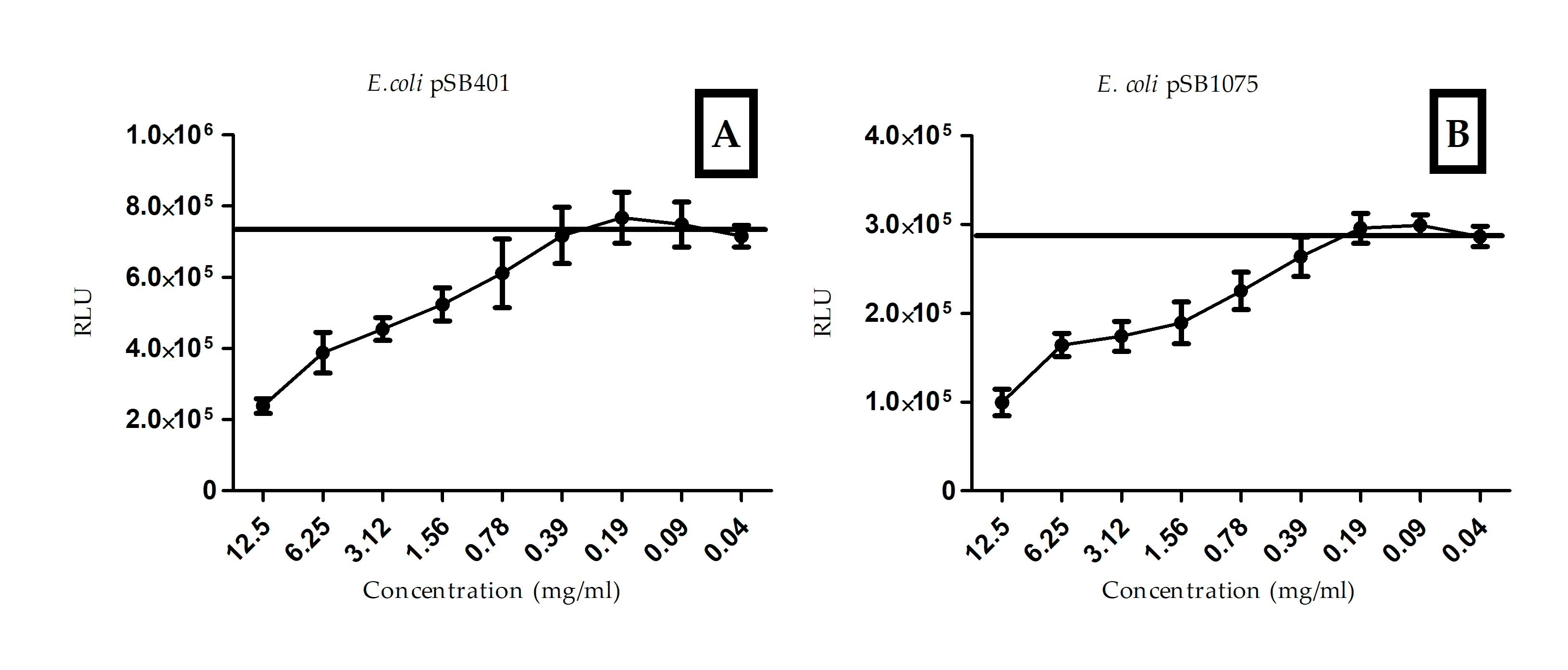
**

**Figure S1**. QS dependent bioluminescence assay of the crude extract of *Streptomyces tendae* VITAKN: **A**) The LuxR-based reporter *E. coli* pSB401 induced by OXO-C6-AHL; B) The LasR-based reporter *E. coli* pSB1075 induced by OXO-C12-AHL. Data is expressed as SD of mean (n = 3). The bioluminescence for the negative control is shown by line.

**Table S1**: Growth pattern of isolate *Streptomyces tendae* VITAKN on different media.

| **Media** | **Growth** | **Aerial mycelium** | **Substrate mycelium** |
| --- | --- | --- | --- |
| ISP1 | Excellent | White | Grey |
| ISP2 | Excellent | Yellowish white | Yellow |
| ISP3 | Moderate | Whitish | Grey |
| ISP4 | Excellent | Whitish | Grey |
| ISP5 | Good | Grey | Grey |
| ISP6 | Moderate | Whitish | Grey |
| ISP7 | Excellent | Grey | Grey |
| Starch Casein Agar | Good | Grey | Grey |
| Nutrient Agar | Moderate | White | Grey |
| Actinomycetes Isolation Agar | Good | White | Grey |

**Table 2.** Biochemical tests for *Streptomyces tendae* VITAKN.

| **Properties** | |
| --- | --- |
| Gram’s stain | + |
| Motility | - |
| Aerial and substrate mycelium | + |
| Surface of the spore | Smooth |
| Colour of aerial mycelium | White |
| Shape of the spores | Oblong |
| Indole production | - |
| Methyl Red test | + |
| Voges Proskauer’s test | - |
| Citrate Utilization test | + |
| Oxidase test | + |
| Catalase test | + |
| Triple Sugar Iron test | k/k |
| Urease test | + |
| Mannitol motility test | - |
| Amylase | - |
| Lipase | + |
| **Utilization of carbon source** | |
| Glucose | ++ |
| Fructose | +++ |
| Maltose | +++ |
| Mannitol | +++ |
| Starch | + |

Notes: Positive (+); Negative (-); k/k – alkaline slant, alkaline butt; Good (+++); Moderate (++); Fair (+)

**
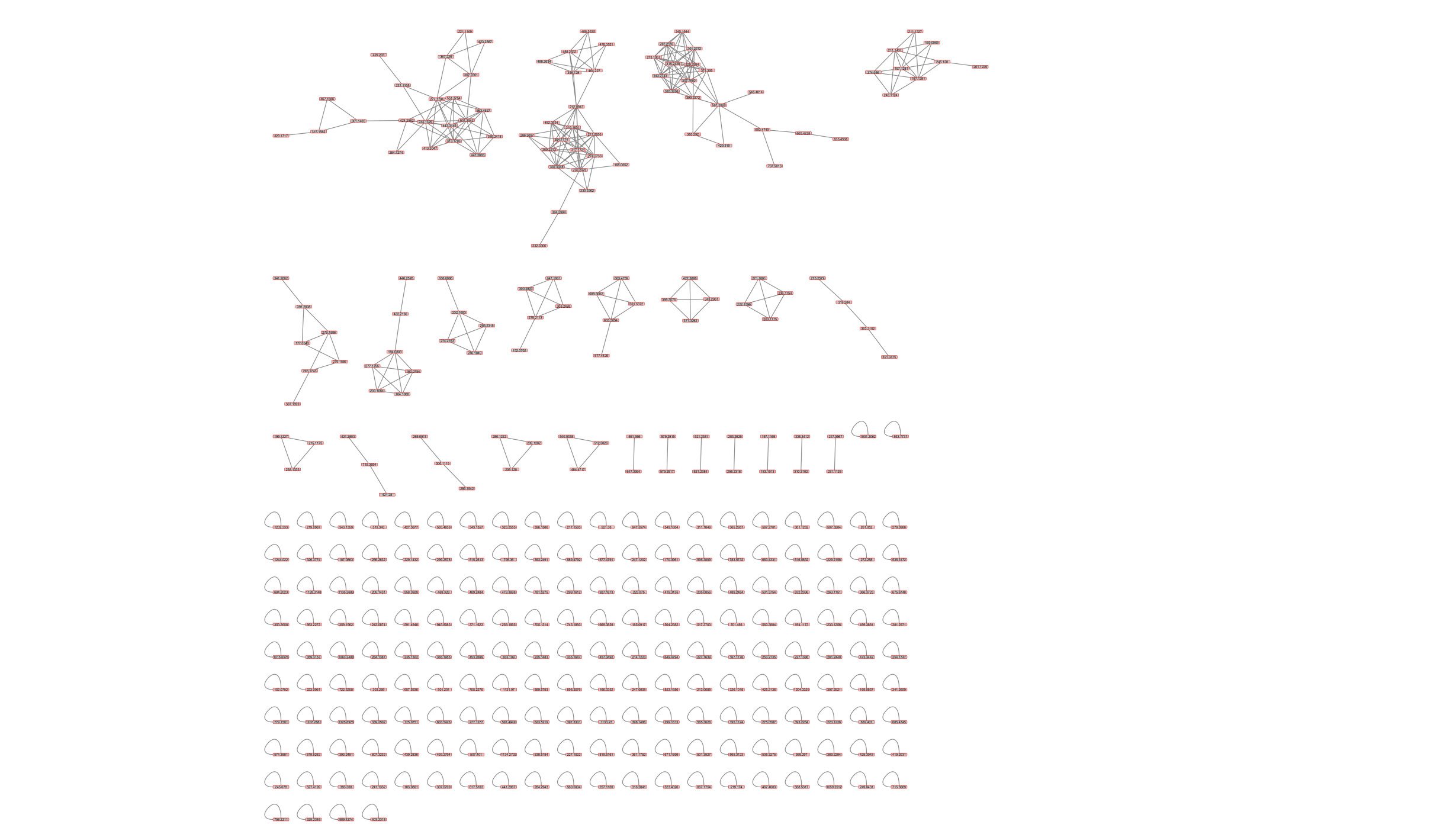
**

**Figure S2**: Generation of molecular network with various clusters produced by crude extract of *Streptomyces tendae* VITAKN by GNPS above similarity score threshold. Nodes highlighted in colored boxes represent parent ions.
